# Epidemiological Characteristics of Scorpionism in West Azerbaijan Province, Northwest of Iran

**DOI:** 10.18502/jad.v14i2.3738

**Published:** 2020-06-30

**Authors:** Samira Firooziyan, Ali Sadaghianifar, Javad Rafinejad, Hassan Vatandoost, Mulood Mohammadi Bavani

**Affiliations:** 1 Department of Medical Entomology and Vector Control, School of Public Health, Tehran University of Medical Sciences, Tehran, Iran; 2 Urmia Health Center, Disease Control Unit, Urmia University of Medical Sciences, Urmia, Iran; 3 Department of Chemical Pollutants and Pesticides, Institute for Environmental Research, Tehran University of Medical Sciences, Tehran, Iran; 4 Department of Medical Entomology and Vector Control, School of Public Health, Urmia University of Medical Sciences, Urmia, Iran

**Keywords:** Scorpion, Scorpionism, West azerbaijan

## Abstract

**Background:**

There are four medically important scorpion species (*Mesobuthus eupeus*, *Mesobuthus caucasicus*, *Androctonus crassicauda* and *Hottentotta saulcyi*) in the West Azerbaijan Province, northwestern Iran. scorpionism is considered as a health problem in this region, because there is no information about scorpion envenomation, this study was designed to study epidemiological characteristics of scorpionism to optimize prevention and treatment of scorpion sting in northwest of Iran.

**Methods:**

All the data from epidemiological surveys completed in West Azerbaijan hospitals over four years (2014–2017) for scorpion victims were collected. This information includes the number of victims, sex, age, signs and symptoms, site of sting, body parts of victims, history of previous sting, the condition of the patient in terms of recovery and death, and the time to receive anti venom, all data were analyzed by the Excel software.

**Results:**

A total of 2718 cases of scorpionism were reported from March 2014 to March 2017 in the study area. The most cases occur in both sexes in the age groups of 25 to 34 years old. In urban areas 40.3% of people and in rural areas 59.7% of them have suffered. The Poldasht and Chaldoran cities, had the most and least incidence respectively.

**Conclusion:**

In this study, the high risk areas in the case of scorpion stings were identified. Results of this study can be used to design preventive programs to educate more people about dangerous areas to prevent scorpion sting and even death.

## Introduction

So far 64 species of scorpions have been reported from Iran belonging to Buthidae (86%), Hemiscorpiidae (9.5%), and Scorpionidae families (4.5%) (1).

Scorpions are venomous animals that can sting people and cause public health problems and sometimes can be fatal (2). The medically important scorpion species in Iran belong to two families of Buthidae and Hemiscorpiidae (2). From the Buthidae family the species of *Mesobuthus eupeus*, *Mesobuthus phillipsii*, *Mesobuthus caucasicus*, *Odontobuthus doriae*, *Odontobuthus bidentatus*, *Orthochirus iranus*, *Buthacus macrocentrus*, *Apistobuthus susanae*, *Compsobuthus matthiesseni*, *Hottentotta saulcyi*, *Hottentotta zagrosensis* and *Hottentotta jayakari* are the most medically relevant scorpion in Iran (2–8). From Hemiscorpiidae the *Hemiscorpius lepturus* and *Hemiscorpius acanthocercus* species are considered as dangerous and deadly scorpion in Iran (7, 9). Buthidae is the largest family in Iran (2). Most medically important scorpion species have been reported from south and south western provinces of Iran, among which Khuzestan is regarded as high risk area (2, 10).

Buthidae species have mostly neurotoxic effect, but Hemiscorpiidae such as *H. lepturus* (local name: Gadim), causes cell death with hemolytic effect cause more death in the country (11–13).

In Iran for scorpion sting treatment polyvalent antivenom is used. This antivenom is prepared by Razi institute against six medically relevant scorpion species: *M. eupeus*, *A. crassicauda*, *O. doriae*, *H. saulcyi*, *H. zagrosensis* and *H. lepturus* (2, 3).

There are four medically important scorpion species: *M. eupeus*, *M. caucasicus*, *A. crassicauda* and *H. saulcyi*: in West Azerbaijan Province, northwestern Iran, among which *M. eupeus* is the most prevalence and *Androctonus crassicauda* is the deadliest (3, 14).

The people most affected by scorpion stings usually live in poor communities where medical resources are often sparse. Scorpion sting is a common health problem all over the world, including Iran. Nearly 50000 cases of scorpion stings have been reported annually from Iran (3, 15). Some studies on scorpionism in Iran shows: the most cases of scorpion stings occurred in rural area in summer season (16–20). The age groups of 10–24 and 25–44 years old are more at risk (17, 20–22). Foot and hand are more frequent bitten by scorpions (16, 20, 22–25).

Scorpion venoms, which are especially lethal in young children, release autonomic nervous system mediators causing myocardial damage, cardiac arrhythmias, pulmonary edema, shock, paralysis, muscle spasms and pancreatitis (26). Early administration of anti-venom is highly effective, together with intensive care support in severe cases. However, the rapid tissue distribution of scorpion venom toxins and their ability to cause early death especially in young children, demands early treatment with anti-venom and full cardio-respiratory support (27).

The true incidence of scorpion sting envenoming is not known because many cases do not seek medical attention. However, it has been estimated that there are approximately 1 million stings per year in the world. In Northern Africa, the Middle East (notably Iran), India and Latin America scorpion stings are an emergent health problem, due to the adaptation of some scorpion species to the urban environment (15).

Since over the past 4 years, deaths from scorpion sting have been reported from West Azerbaijan province and Scorpion sting is a public health problem in this region of Iran. There is no information about epidemiological characteristics of scorpionism in this area. Therefore, this study was designed to describe epidemiological characteristics of scorpion envenomation in West Azerbaijan Province, northwest of Iran to optimize prevention and treatment through community awareness.

## Materials and Methods

### Study area

**Fig. 1.**
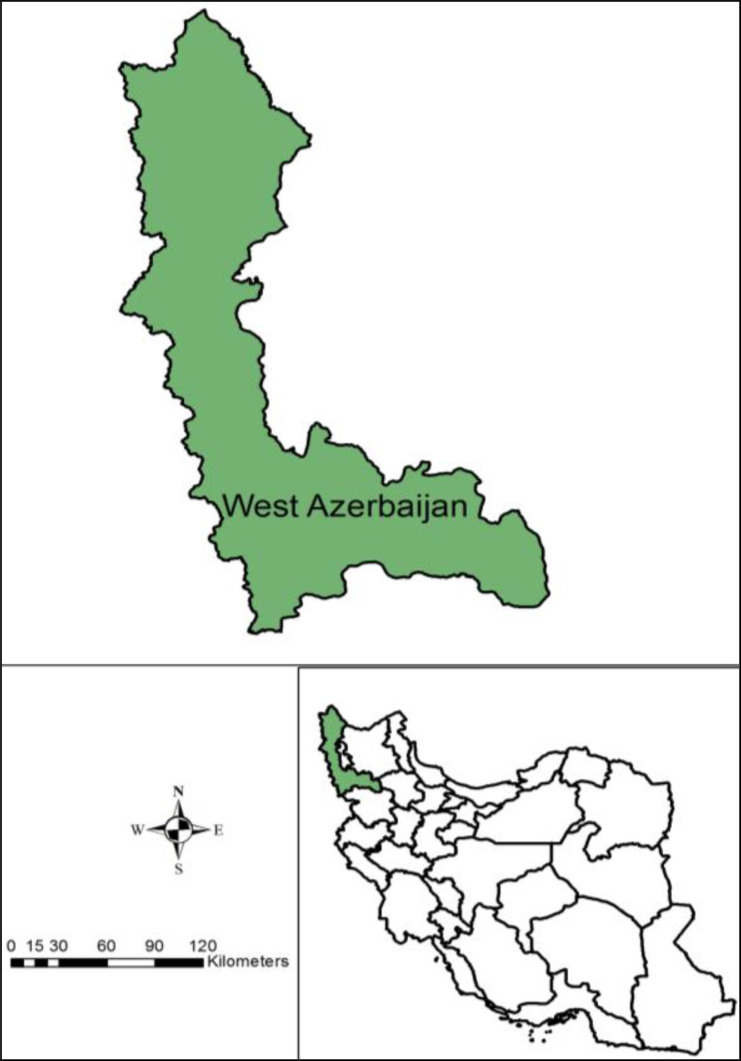
The status of study area, West Azerbaijan Province in Iran

West Azerbaijan Province is located in the northwest of Iran (Fig. 1), bordering Turkey, Iraq and Azerbaijan’s Nakhchivan Autonomous Republic, as well as the provinces of East Azerbaijan, Zanjan and Kurdistan. It is separated from Armenia by Turkey’s short border with the Azerbaijan Republic. This province covers an area of 39,487km^2^, or 43,660 km^2^ including Lake Urmia, between 37.5528° N and 45.0759° E. In 2012 the province had a population of about 3 million (estimate). The capital and largest city of the province is Urmia. The people of this province are active in agriculture and animal husbandry. For this reason, the province has been selected to examine the status of scorpion sting and important medical species.

### Data collection

The data from epidemiological surveys completed in West Azerbaijan hospitals over four years for scorpion victims were collected. This information includes the number of victims, sex, age, signs and symptoms, body parts of victims, time of sting, history of previous sting, the condition of the patient in terms of recovery and death and the time to receive anti venom. The above information was analyzed by the Excel software. A spatial distribution map of scorpionism was introduced using GIS 4.2.

## Results

**Fig. 2.**
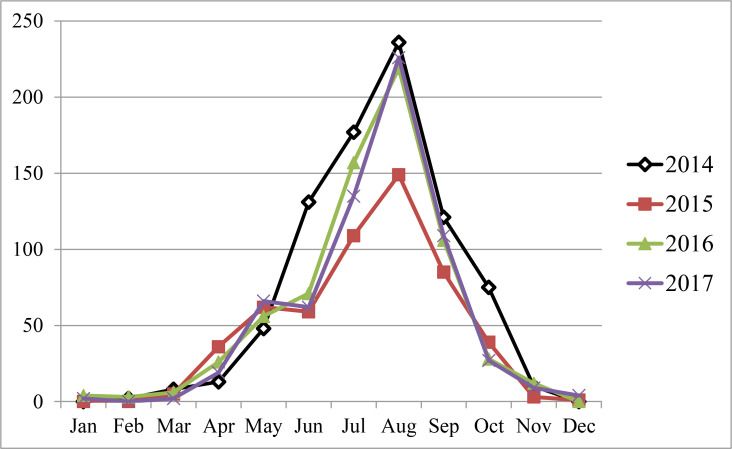
Monthly scorpion sting cases, West Azerbaijan, northwest of Iran, 2014–2017 (X: Month, Y: Scorpion sting cases)

From March 2014 to March 2017, a total of 2718 cases of scorpion sting from West Azerbaijan Province were recorded. The largest number (821 cases) was in 2014 and the lowest (548 cases) in 2015 (Fig. 2). Many scorpion stings occurred during the hot months of May to September with a peak in August (Fig. 2).

In these four years, 53.6% of cases occurred in men and 46.4% in women. In males, the most cases were in the age group of 25 to 34 years old and the least cases were in the age group of 5 to 9 years old (Table 1), while in the females, the highest number of victims was in the age group of 25 to 34 years, and the smallest in the age group of 0–4 years old (Table 2). Therefore, the most cases of scorpion sting in both sexes were in the age group of 25 to 34 years old. 40.3% of stung people in urban areas and 59.7% in rural areas were reported. Among 2718 patients, in 80 cases (2.9%) the head and neck, in 1364 cases (50.2%) the hands, in 1069 cases (39.3%) the leg and in 205 cases the trunk were bitten (Table 3).

**Table 1.** Stung persons classified by age group (males)

**Years**	**0–4**	**5–9**	**10–14**	**15–24**	**25–34**	**35–44**	**45–54**	**55–64**	**>65**	**Total**
**2017**	13	6	17	56	68	57	41	28	19	**305**
**2016**	8	19	20	61	76	55	42	20	12	**313**
**2015**	12	8	13	48	61	46	46	13	15	**262**
**2014**	7	13	16	83	93	66	56	26	20	**380**
**Total**	40	46	66	248	298	224	185	87	66	**1260**
**%**	3.2	3.7	5.2	19.7	23.6	17.8	14.7	6.9	5.2	**100**

**Table 2.** Stung persons classified by age group (females)

**Years**	**0–4**	**5–9**	**10–14**	**15–24**	**25–34**	**35–44**	**45–54**	**55–64**	**>65**	**Total**
**2017**	24	15	20	77	82	66	34	20	18	**356**
**2016**	13	19	27	82	96	58	41	22	17	**375**
**2015**	13	12	14	54	77	47	44	10	15	**286**
**2014**	19	11	18	103	111	90	45	22	22	**441**
**Totally**	69	57	79	316	366	261	164	74	72	**1458**
**%**	4.7	4	5.4	21.7	25.1	17.9	11.2	5.1	4.9	**100**

**Table 3.** Stung persons according to body part

**Years**	**Head and neck**	**Hand**	**Leg**	**Trunk**	**Total**
**2017**	17	330	258	56	**661**
**2016**	15	344	286	43	**688**
**2015**	19	254	228	47	**548**
**2014**	29	436	297	59	**821**
**Total**	80	1364	1069	205	**2718**
**%**	3	50.2	39.3	7.5	**100**

In this study, the most cases of scorpion stings including 1263 cases (46%) occurred: at 00:00 to 6:00AM and the lowest cases including 456 cases (16.8%) occurred at 12:00 AM to 6:00PM (Table 4). 8 percent of the cases (n= 217) had previous history of sting and 2.2% (n= 61) had used previous scorpion anti venom in the past four years.

**Table 4.** Time of scorpion stings in stung persons

**Years**	**0–6**	**6–12**	**12–18**	**18–24**	**Total**
**2017**	203	153	163	142	**661**
**2016**	248	166	122	152	**688**
**2015**	204	115	106	123	**548**
**2014**	608	65	65	83	**821**
**Totally**	1263	499	456	500	**2718**
**%**	46.5	18.3	16.8	18.4	**100**

**Fig. 3.**
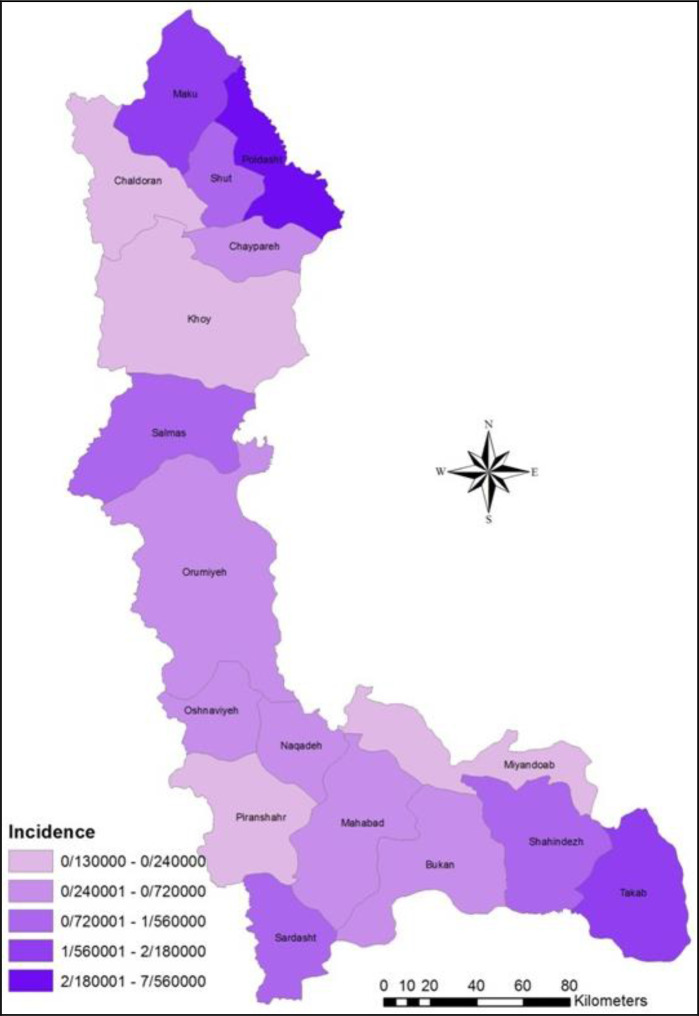
Spatial distribution of incidence of scorpion sting from West Azerbaijan of Iran, 2014–2017

The time of treatment between sting and anti-venom injection in 70.3% of patients were less than 6 hours, in 8% of patients were 6 to 12 hours, in 6.4% of patients lasted more than 12 hours and 15.3% of patients did not receive anti venom (Table 5). The percentage of recovered cases were 99.93% and the deaths due to scorpion sting were 0.07% (2 out of 2718 cases), of which two deaths were observed in the last two years (one case in 2016 and another in 2017). The average incidence of scorpionism per 1000 people was calculated and Poldasht and Chaldoran counties had the most and least incidence (Table 6, Fig. 3).

**Table 5.** Time of anti-venom injection after sting

**Years**	**Without**	**<6**	**6–12**	**>12**	**Total**
**2017**	72	480	48	61	**661**
**2016**	78	501	65	44	**688**
**2015**	145	338	31	34	**548**
**2014**	120	591	74	36	**821**
**totally**	415	1910	218	175	**2718**
**%**	15.3	70.3	8.0	6.4	**100**

**Table 6.** The average incidence of scorpionism per 1000 people in different areas

**Years**	**2017**	**2016**	**2015**	**2014**	**Totally**	**%**	**per1000**
**Naghadeh**	16	17	14	44	91	3.35	0.71
**Miandoab**	3	10	12	36	61	2.24	0.22
**Mahabad**	43	47	31	46	167	6.14	0.71
**Maku**	38	63	33	73	207	7.62	2.18
**Shoot**	50	8	0	7	65	2.39	1.17
**Shahin dej**	21	26	29	44	1120	4.42	1.30
**Salmas**	48	103	91	66	308	11.33	1.57
**Sardasht**	64	35	27	54	180	6.62	1.51
**Khoy**	15	28	11	22	76	2.80	0.22
**Chaldoran**	1	0	5	0	6	0.22	0.13
**Chaipareh**	6	1	8	3	18	0.66	0.40
**Takab**	51	48	14	58	171	6.29	2.12
**Piranshahr**	7	18	5	3	33	1.21	0.24
**Poldasht**	74	107	92	46	319	11.74	7.56
**Bukan**	21	19	23	38	101	3.72	0.40
**Oshnavieh**	23	4	4	10	41	1.51	0.55
**Urmia**	180	154	149	271	754	27.74	0.72
**Total**	661	688	548	821	2718	100	0.83

## Discussion

Scorpion stings are considered as one of the most medically important problem in Iran. There are 64 species of scorpions belonging to tree families (Buthidae, Hemiscorpiidae and Scorpionidae) in Iran (1).

Four medically important species of scorpions including: *M. eupeus*, *M. caucasicus*, *A. crassicauda* and *H. saulcyi* have been reported from West Azerbaijan Province and cause public health problems in this region. *M. eupeus* has been captured in the most parts of northwest corner of Iran (3).

Scorpionism is a common health problem all over the world, yet neglected. The true incidence of scorpion sting envenoming is not known because many cases do not seek medical attention. However, it has been estimated that there are approximately 1 million stings per year. In Mexico, Tunisia, Brazil and Khuzestan, south-west Iran, 250000, 40000, 37000 and 25000 stings were reported in 2005 (15). About 37535 scorpion stings were registered in 2009 and in comparison with cases of 2002, we observed notably increasing on incidence of scorpion sting in Iran (6, 18). Although scorpion sting is a public health problem in the West Azerbaijan Province but there is a big gap of information on scorpionism in this region.

Our results showed that over four last years (2014–2017), a total of 2718 cases of scorpion stings have been recorded from West Azerbaijan. In this region, the age group of 25 to 39 is more at risk and the health system in community education should address this age group, while some studies have suggested that most of the victims of scorpionism are younger than 25 years old (18, 19, 28).

Results of some studies like our results shows that the age of the most of stung persons are more than 25 years old (17, 21, 22). This age group is more active in farming, ranching and gardening. Because of their greater activity, they are more likely to be contacted by scorpions. The health system in community education should address this age group.

Our results showed that 53.6% of cases occurred in men and 46.4% in women that scorpion sting among males sex were 7.2% more than in females. According to reports from the World Health Organization and some studies, the same result was reported (15, 29, 30). While in some other studies unlike our results scorpion stings in females were more than in males (22, 31, 32).

However, in West Azerbaijan Province, because men are more active than women and most of the farming and gardening work is done by men so they are more at risk in the case of scorpion stings, therefore men should be given more training in order to prevent further scorpion sting.

In this study, similar to some other studies, hand, leg, trunk, head and neck, respectively, are more likely to be stung by scorpions (18, 29, 33–36). Like our results in some studies foot and hand have been more frequent bitten by scorpions (16, 20, 22–25). This may be because most people do not take protective measures like using gloves and boots while working in the fields and gardens. Therefore, these organs are easily exposed to scorpion sting. We have to educate people to carefully examine shoes before wearing them and to use gloves and safety shoes when working in an open environment and do not move stones for no reason.

In this study, about 15.3% of the victims didn’t receive antivenin. As most cases occur in the village (60%), the villagers should be informed that antivenin is available in hospitals and health centers. because more sting occur per day, they can easily get antivenin from the health center for free.

Our results showed that many scorpion stings occurred during the hot months of May to September with a peak in August. The results of some studies on scorpion envenomation in Iran, in this case are similar to our results that the peak of scorpion stings occurs in hot month of the year (16–20). This can be for two reasons: In the hot months of the year the scorpions are more active and also people’s activities such as agriculture, gardening and other activities are increasing. These makes people more likely to encounter scorpions and bitten by them.

In our study 99.93% of stung people were recovered and 0.07% of them died. Because the species *M*. *eupeus* has a wide distribution in this area (3). Likely this species caused the most scorpion stings in this region. *Androctonus crassicauda* is regarded as a deadly scorpion in Iran (2), and this species has been reported from this area (3). This black species is probably responsible for the death from the scorpion sting in this corner of Iran.

## Conclusion

In this study, the high risk areas of the province were identified as scorpion sting by using spatial distribution. The results of this study can be used to design preventive programs, to educate more people about the important areas of the province’s area at the risk of scorpion sting, and the prevention of scorpion sting and even death. Because over the past four years, death has been reported due to scorpion sting in the province, people should be informed that when working on open spaces, they should use safety devices for their hands and legs so that the scorpions cannot sting them.
